# Nomogram based on the advanced lung cancer inflammation index and other relevant clinical factors for patients with cervical squamous cell carcinoma undergoing concurrent chemoradiotherapy

**DOI:** 10.1186/s12885-025-14465-6

**Published:** 2025-07-01

**Authors:** Xiao-Chun Wang, Xue-Lian Xu, Shou-Yu Wang, Hao Cheng, Peng-Fei Yan, Ming-Yu Yang, Ke-Chen

**Affiliations:** 1 Department of Radiotherapy Oncology, The First Affiliated Hospital of Xinxiang Medical University, Xinxiang, Henan 453100 China; 2 Department of Pediatric Surgery, The First Affiliated Hospital of Xinxiang Medical University, Xinxiang, Henan 453100 China

**Keywords:** Advanced lung cancer inflammation index, Cervical squamous cell carcinoma, Risk stratification analysis, Concurrent chemoradiotherapy, Nomogram

## Abstract

**Background and objective:**

This study aims to explore the association between the advanced lung cancer inflammation index (ALI), the adjusted Charlson Comorbidity Index (ACCI), and other relevant clinical factors and the prognosis of patients with cervical squamous cell carcinoma (CSCC) who undergoing concurrent chemoradiotherapy, and to construct a corresponding prognostic model.

**Methods:**

A total of 243 patients with CSCC who undergoing concurrent chemoradiotherapy between January 2017 and December 2023 were included in this study. Univariate and multivariate Cox regression analyses were conducted to identify independent prognostic factors influencing progression-free survival (PFS) and overall survival (OS). These independent prognostic factors were subsequently utilized to construct two nomograms, which were then subjected to a comprehensive series of validations. Ultimately, a risk stratification framework was developed to evaluate the prognostic outcomes of patients across varying risk categories.

**Results:**

ALI, ACCI, American Joint Committee on Cancer (AJCC) stage, and tumor volume were identified as independent predictors of PFS and OS (all *P* < 0.05). Based on these independent clinical factors, we developed two distinct nomograms for the prediction of progression-free survival (PFS) and overall survival (OS), respectively. In the training cohort, the C-indexes of the model for PFS and OS were 0.743 and 0.741, respectively; while the corresponding C-indexes were 0.735 and 0.728 in the validation cohort. Following an extensive series of validations, the newly developed nomogram models demonstrated superior performance compared to the traditional AJCC staging system. Based on the total risk points derived from the nomograms, we stratified all patients into three risk subgroups: high-risk, medium-risk, and low-risk. Patients in three distinct risk subgroups exhibited significantly different survival outcomes.

**Conclusion:**

ALI has significant value for predicting PFS and OS of CSCC patients who have undergone concurrent chemoradiotherapy. The newly developed nomogram models based on ALI demonstrates robust performance and offers a valuable reference for personalized treatment strategies.

**Supplementary Information:**

The online version contains supplementary material available at 10.1186/s12885-025-14465-6.

## Introduction

Cervical cancer is the fourth most common cancer in terms of both incidence and mortality in women, with an estimated 660,000 new cases and 350,000 deaths worldwide in 2022 [1]. The disease is the most common cancer type in 25 countries and the leading cause of cancer death in 37 countries, mainly in sub-Saharan Africa as well as South America and Southeastern Asia [1]. Squamous cell carcinoma (SCC) is the most common histological type of cervical cancer [2]. concurrent chemoradiotherapy is one of the important treatment methods for cervical squamous cell carcinoma, especially for patients with locally advanced disease [3]. However, the prognosis of patients with cervical squamous cell carcinoma who receive concurrent chemoradiotherapy varies significantly [4]. The most common cause of cervical cancer is persistent infection caused by the sexually transmitted human papilloma virus(HPV) [5, 6]. Other factors that contribute to the incidence of cervical cancer include geography, traditional practices and beliefs, the screening levels, socioeconomic status, healthcare access, public awareness, use of oral contraceptives, smoking and coinfection with Human Immunodeficiency Virus (HIV) [7]. Due to multiple factors such as higher HPV vaccination rates, better economic foundations, and more standardized treatment methods, the prognosis of cervical cancer in high-income countries is better than that in Low- and Middle-Income Countries (LMICs). In 2018, 84–90% of global cervical cancer deaths occurred in LMICs such as South Africa, India, China and Brazil [8]. In China, the age-standardized incidence rate of cervical cancer has shown a significant upward trend, which is a major public health issue [9]. Thus, precise prognosis prediction can aid clinicians in making informed treatment decisions and providing comprehensive patient consultations.

In the past, the prognosis of cervical cancer was mainly evaluated based on AJCC staging [10] or International Federation of Gynecology and Obstetrics (FIGO) staging [11]. While the AJCC staging system serves as a crucial predictive tool, it predominantly focuses on the extent of anatomical invasion and spatial dissemination of the tumor. Consequently, it overlooks several significant patient-related factors, including demographic characteristics (such as age, BMI, and marital status), treatment modalities, inflammatory and nutritional markers, socioeconomic status, lifestyle habits, psychological well-being, and comorbidities. Nomograms are widely used for cancer prognosis [12], primarily because of their ability to reduce statistical predictive models into a single numerical estimate of the probability of an event, such as death or recurrence, that is tailored to the profile of an individual patient [13].

In recent years, a large number of clinical studies [14–16] have shown that inflammatory indices such as ALI, as independent clinical factors, have significant clinical significance in the prognosis assessment of tumor patients. ALI is a composite index that integrates body mass index (BMI), serum albumin level, and neutrophil-to-lymphocyte ratio (NLR). This index serves as an effective indicator of both systemic inflammation and nutritional status. In 2013, Jafri et al. first introduced ALI as a novel prognostic biomarker for non-small cell lung cancer (NSCLC) patients [17]. Subsequently, ALI has been validated in many different treatments and diseases of NSCLC [18], and small cell lung cancer (SCLC) [19]. The prognostic ability of ALI is superior to other inflammation/nutrition-based indicators in all lung cancer patients [16]. Moreover, studies have shown that in various other cancer entities, including hepatocellular carcinoma [20], Gastrointestinal Cancers [21], squamous head and neck cancer [22], a significant reduction in ALI will have an adverse impact on survival rates and have a better prognostic predictive value for patients. However, the impact of ALI on patients undergoing concurrent chemoradiotherapy for cervical squamous cell carcinoma (CSCC) remains to be elucidated. In light of the aforementioned considerations and the requirements of personalized medicine, this study aims to investigate the predictive value of ALI and integrate it into a nomogram model to enhance the prognosis prediction for CSCC.

## Materials and methods

### Materials

**Fig. 1:**
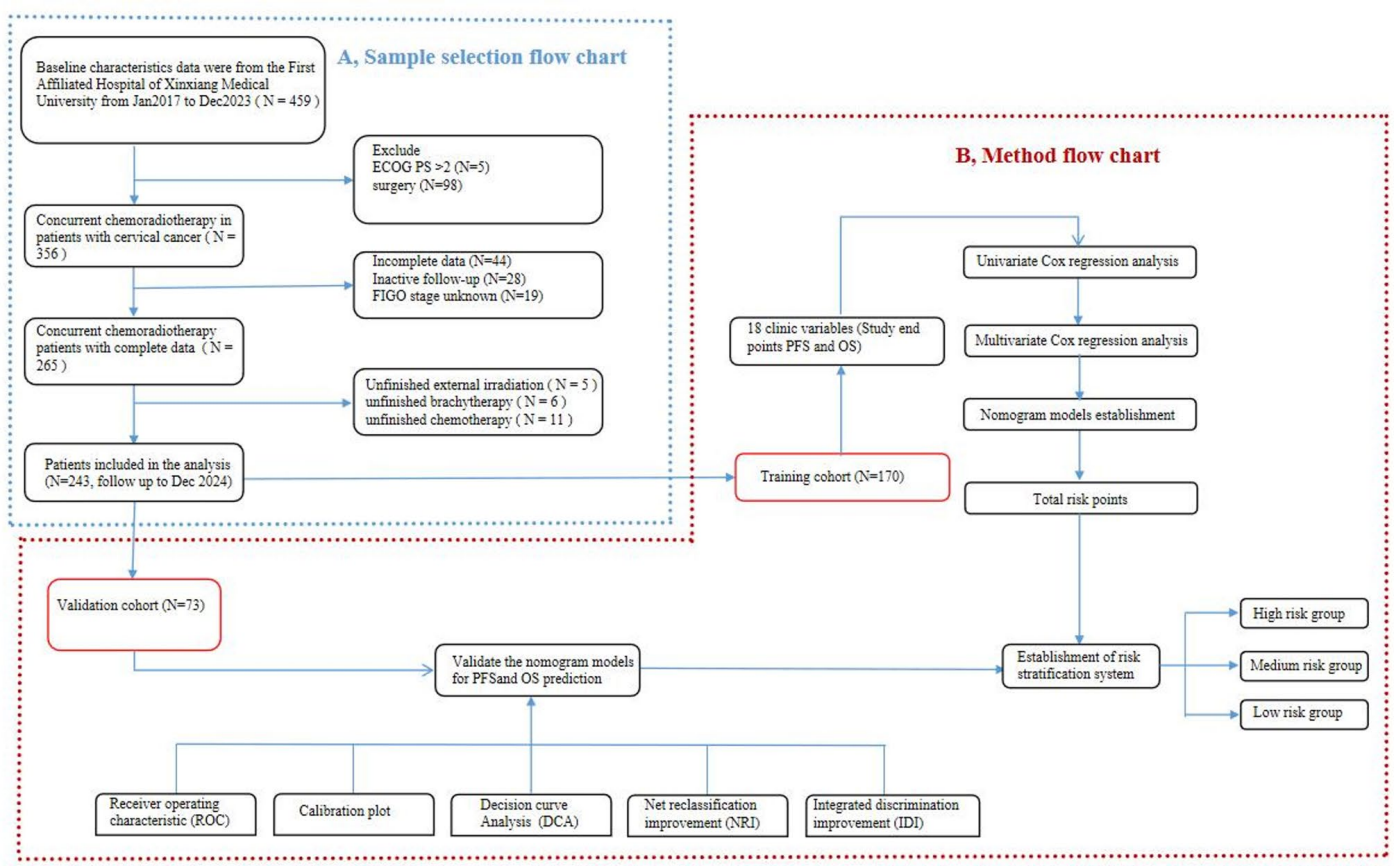
Study workflow. Diagram **A** illustrates the sample selection steps, and Diagram **B** depicts the statistical analysis process. Abbreviations: AJCC, American Joint Committee on Cancer; PFS, Progression-Free Survival; OS, Overall survival

A total of 243 CSCC who undergoing concurrent chemoradiotherapy in the First Affiliated Hospital of Xinxiang Medical University from January 2017 to December 2023 were enrolled. The cases obtained according to the inclusion and exclusion criteria are as follows. The inclusion criteria included: (1) patients with cervical cancer confirmed by pathology as squamous cell carcinoma; (2) active follow-up. Exclusion criteria included: (1) those who underwent surgical treatment, (2) Eastern Cooperative Oncology Group performance status score (ECOG PS) > 2, (3) incomplete data, (4) AJCC stage unknown, (5) inactive follow-up for more than 30 days, (6) the whole radiotherapy process was not completed according to the radiotherapy plan, (7) concurrent chemotherapy was not received. The flow chart is displayed in Fig. 1A. Chemotherapy regimens comprised platinum-based compounds and taxane derivatives. The 9th edition of the AJCC staging system was utilized.

All cases were randomly allocated into training and validation groups at a 7:3 ratio using SPSS 20.0. The nomogram was developed based on the training group, while its validation was performed using the validation group. A total of 17 clinical variables related to CSCC patients who underwent concurrent chemoradiotherapy were included in the analysis. These variables comprised: age at diagnosis, AJCC stage, SCCA levels, tumor volume, BMI platelet-to-lymphocyte ratio (PLR), platelet-to-albumin ratio (PAR), NLR, lymphocyte-to-monocyte ratio (LMR), prognostic nutritional index (PNI), systemic immune-inflammation index (SII), ALI, menopausal status, hypertension, diabetes, adjusted Charlson comorbidity index (ACCI), and systemic inflammation score (SIS). We utilized the X-tile software to determine the optimal cut-off values for tumor volume, PLR, NLR, PNI, SII, and ALI. These cut-off values were subsequently employed to transform the aforementioned continuous variables into categorical variables, as detailed in Tables 2 and 3.

The flowchart outlining the process for establishing and validating the nomogram to predict outcomes in CSCC who undergoing concurrent chemoradiotherapy is shown in Fig. 1B. Tables S1 provide detailed calculation methods for PLR, PAR, NLR, LMR, SII, BMI, ALI, and SIS. Table S2 outlines the scoring methodology utilized for the Assessment of ACCI.

The radiotherapy received by patients with cervical cancer encompasses external beam radiotherapy and brachytherapy, with a total equivalent two-dimensional dose (EQD2) surpassing 85 Gy, and Concurrent chemotherapy mainly consists of cisplatin, with more than 4 cycles. The PFS and OS were considered as study endpoints.

### Statistical analytics

R software (version 4.2.2) and SPSS 26.0 were used for statistical analyses. To compare baseline characteristics between the training and validation cohorts, the chi-square test and independent samples t-test were applied. Univariate Cox regression analysis was performed to evaluate the association between clinical variables and PFS or OS. Variables with *P* < 0.05 were included in the multivariate Cox proportional hazards model, and stepwise regression was used to identify independent prognostic factors.

Based on these independent prognostic variables, a nomogram was constructed using the “rms” package in R, which was built on a multivariate Cox model. The coefficients of each significant variable were scaled into points to calculate a total risk score, and the probability of events at different time points (PFS or OS) was estimated. No machine learning techniques were used in the construction process; instead, a traditional statistical modeling approach was adopted to ensure interpretability and clinical applicability.

In this study, R software was employed to compute calibration curves, the concordance index (C-index), receiver operating characteristic curves (ROC), decision curve analysis (DCA), integrated discrimination improvement index (IDI), and net reclassification improvement index (NRI). The ROC curve was utilized to assess the performance of the classification model, illustrating its comprehensive evaluation of the true positive rate and false positive rate across various thresholds. DCA was employed to validate the clinical utility of the models. Calibration plots were used to evaluate the accuracy of the model’s predicted probabilities, ensuring that the predicted outcomes align with the actual data distribution. The IDI and NRI showed the improvement of the new nomogram over the traditional AJCC staging system.

Ultimately, a novel risk stratification was established based on the total risk points of the nomogram, and all patients were classified into low-, medium-, and high-risk groups using X-tile software. Log-rank tests and Kaplan-Meier survival curves were adopted to compare the survival durations of different risk stratification groups.

## Results

### Characteristics of patients

A total of 243 CSCC who undergoing concurrent chemoradiotherapy were included in this study and randomly divided into a training group (*n* = 170) and a validation group (*n* = 73) at a ratio of 7:3. All patients received a standardized concurrent chemoradiotherapy regimen. The median PFS of the patients amounted to 35 months, while the median OS reached 46 months. The detailed clinical characteristics of the cases incorporated into the study are presented in Table 1. Based on the data in Table 1, with the exception of menopausal status, no significant statistical discrepancies were observed in other clinical factors between the training group and the validation group. The median age of the patients was 56 years. The tumor stage was mostly stage II (53.4%). The median SCCA value was 15, and the median tumor volume was 19.5.The proportion of menopausal patients is 68%, that of patients with hypertension is 32%, and that of patients with diabetes is 14.5%. In this study, the multiple collinearity of prognostic variables in CSCC who undergoing concurrent chemoradiotherapy was detected using SPSS software. The variance inflation factor (VIF) of all variables was less than 5, indicating that there was no significant multiple collinearity among the prognostic variables (Table S3). In predicting PFS, the C-index values for ALI, albumin, BMI, NLR, PLR, PAR, LMR, PNI, and SII were 0.621, 0.554, 0.572, 0.561, 0.555, 0.536, 0.561, 0.570, and 0.540, respectively. For OS, the corresponding C-index values were 0.628, 0.560, 0.567, 0.553, 0.545, 0.545, 0.563, 0.566, and 0.550. These results indicate that ALI consistently showed the highest discriminatory ability for both PFS and OS among the biomarkers.

**Table 1:** Clinical information of CSCC who undergoing concurrent chemoradiotherapy in the training group and the validation group

Characteristics	All Patients	Training cohort	Validation cohort	*P*
	(*n* = 243)	(*n* = 170)	(*n* = 73)	
	*N* (%)	*N* (%)	*N* (%)	
**Age at diagnosis (years)**				0.288
Median (range)	56(29–90)	56 (29–81)	56 (29–90)	
**AJCC_Stage**				0.164
I	21 (8.6%)	18(10.5%)	3 (4.1%)	
II	130 (53.4%)	94 (55.2%)	36 (49.3%)	
III	57 (23.4%)	35 (20.5%)	22 (30.1%)	
IV	35 (14.4%)	23 (13.5%)	12 (16.4%)	
**SCCA**				0.521
Median (IQR)	15 (6.5–26)	15 (6.2–26.2)	11 (6.5–23.3)	
**Tumor_volume**				0.536
Median (IQR)	19.5 (10.2–46.3)	19.15 (9.2–45)	21.9 (11.1–41.4)	
**BMI (kg/m2)**				0.09
Median (IQR)	23.6 (22.0-25.8)	23.6 (22.1–25.9)	23.1 (21.5–25.0)	
**PLR**				0.646
Median (IQR)	183.2 (124.2-255.4)	183.1 (119.1-263.6)	185.5 (127.8–235)	
**PAR**				0.789
Median (IQR)	6.1 (4.6–7.5)	6.05 (4.5–7.4)	6.2 (4.6–7.4)	
**NLR**				0.375
Median (IQR)	3 (1.9–4.6)	2.9 (1.8–4.4)	3 (2.1–4.9)	
**LMR**				0.809
Median (IQR)	5.6 (3.8–7.2)	5.65 (3.7–7.3)	5.4 (3.8–6.9)	
**PNI**				0.246
Median (IQR)	48.6 (45.4–52.2)	48.8 (45.2–52.0)	48.6 (45.2–52.5)	
**SII**				0.362
Median (IQR)	770.9 (441.1-1233.4)	739.15 (4400.8-1157.2)	826.7 (434.6-1341.1)	
**ALI**				0.738
Median (IQR)	33.3 (20.8–53.4)	34.25 (21.1–54.9)	32.2 (19.5–49.9)	
**Menopause**				0.049
No	78 (32%)	48 (28.2%)	30 (41%)	
Yes	165 (68%)	122 (71.8%)	43 (59%)	
**Hypertension**				0.304
No	165 (68%)	112 (65.8%)	53 (72.6%)	
Yes	78 (32%)	58 (34.2%)	20 (27.4%)	
**Diabetes**				0.847
No	208 (85.5%)	170 (65.9%)	62 (84.9%)	
Yes	35 (14.5%)	88 (34.1%)	11 (15.1%)	
**ACCI**				0.297
< 4	198 (81.5%)	139 (81.7%)	59 (80.8%)	
≥ 4	45 (18.5%)	31 (18.3%)	14 (19.2%)	
**SIS**				0.906
0	104 (42.7%)	72 (42.3%)	32 (43.8%)	
1	95 (39%)	66(38.8%)	29 (39.7%)	
2	44 (18.1%)	32 (18.8%)	12 (16.4%)	
**PFS (months)**				0.312
Median (range)	35 (1–96)	34.5 (1–96)	36 (1–88)	
**OS (months)**				0.452
Median (range)	46 (3–96)	46 (3–96)	45.5 (3–88)	

*Abbreviations* ACCI, age-adjusted Charlson comorbidity index; ALI, advanced lung cancer inflammation index, AJCC, American Joint Committee on Cancer; BMI, body mass index; CSCC, cervical squamous cell carcinoma; IQR, interquartile range; LMR, lymphocyte-to-monocyte ratio; NLR, neutrophil-to-lymphocyte ratio; OS, overall survival; PAR, platelet-to-albumin ratio; PFS, progression-free survival; PLR, platelet-to-lymphocyte ratio; PNI, prognostic nutrition index; SCCA, squamous cell carcinoma antigen; SII, systemic immune-inflammation index; SIS, systemic inflammation score

### Univariate and multivariate Cox regression results

Univariate and multivariate Cox regression analyses were conducted to identify prognostic factors for PFS and OS. Univariate analysis revealed nine potential factors associated with PFS in CSCC patients undergoing concurrent chemoradiotherapy: AJCC stage, tumor volume, ACCI, PLR, PAR, NLR, PNI, SII, and ALI (Table 2). Additionally, six variables were identified as potentially influential for OS in this patient population: AJCC stage, tumor volume, ACCI, NLR, SII, and ALI (Table 3). Multivariate Cox proportional hazards regression analysis indicated that ALI, ACCI, AJCC stage, and tumor volume were independent predictors of both PFS and OS (all *P* < 0.05). Based on these independent predictors, a nomogram model was developed to predict PFS and OS in CSCC patients receiving concurrent chemoradiotherapy.

**Table 2:** Univariate and multivariate analyses in CSCC who undergoing concurrent chemoradiotherapy for PFS

Characteristics	Univariate analysis	*P*	Multivariate analysis	*P*
	HR (95% CI)		HR (95% CI)	
**Age (years)**	1.026 (0.998–1.054)	0.071		
**Menopause**				
No	Reference			
Yes	1.727 (0.822–2.629)	0.149		
**SCCA**	1.006 (0.991–1.021)	0.463		
**Hypertension**				
No	Reference			
Yes	1.449 (0.764–2.751)	0.256		
**Diabetes**				
No	Reference			
Yes	0.914 (0.384–2.178)	0.839		
**AJCC stage**				
I- II	Reference		Reference	
III	1.573 (0.672–3.517)	0.309	1.205 (0.510–2.848)	0.671
IVA	1.716 (1.562–6.671)	**0.002**	2.528 (1.187–5.386)	**0.016**
**Tumor Volume (cm** ^**3**^ **)**				
< 21.1	Reference		Reference	
≥ 21.1	1.952 (0.879–4.104)	**0.040**	3.175 (1.402–7.192)	**0.006**
**ACCI**				
< 4	Reference		Reference	
≥ 4	1.689 (1.165–2.447)	**0.001**	2.372 (1.158–2.856)	**0.018**
**BMI (kg/m** ^**2**^ **)**	1.039 (0.946–1.140)	0.428		
**PLR**				
< 178.9	Reference		Reference	
≥ 178.9	2.002 (1.031–3.895)	**0.040**	1.721 (0.860–3.443)	0.125
**PAR**	1.110 (0.995–1.239)	0.061		
**NLR**				
< 3.10	Reference		Reference	
≥ 3.10	2.198 (1.167–4.141)	**0.015**	1.029 (0.412–2.572)	0.951
**LMR**	0.962 (0.864–1.071)	0.476		
**PNI**				
≥ 51.5	Reference		Reference	
< 51.5	2.308 (1.060–5.025)	**0.035**	1.484 (0.650–3.384)	0.349
**SII**				
< 656.4	Reference		Reference	
≥ 656.4	2.409 (1.216–4.773)	**0.012**	1.096 (0.384–3.132)	0.864
**ALI**				
> 59.2	Reference		Reference	
30.2–59.2	1.610 (0.617–4.202)	0.331	1.606 (0.613–4.202)	0.335
< 30.2	2.586 (1.033–6.474)	**0.042**	4.433 (1.540–12.758)	**0.006**
**SIS**				
0	Reference			
1	1.305 (0.651–2.615)	0.453		
2	1.574 (0.666–3.722)	0.302		

*Abbreviations* ACCI, age-adjusted Charlson comorbidity index; ALI, advanced lung cancer inflammation index, AJCC, American Joint Committee on Cancer; BMI, body mass index; CSCC, cervical squamous cell carcinoma; LMR, lymphocyte-to-monocyte ratio; NLR, neutrophil-to-lymphocyte ratio; PAR, platelet-to-albumin ratio; PFS, progression-free survival; PLR, platelet-to-lymphocyte ratio; PNI, prognostic nutrition index; SCCA, squamous cell carcinoma antigen; SII, systemic immune-inflammation index; SIS, systemic inflammation score

**Table 3:** Univariate and multivariate analyses in CSCC who undergoing concurrent chemoradiotherapy for OS

Characteristics	Univariate analysis	*P*	Multivariate analysis	*P*
	HR (95% CI)		HR (95% CI)	
**Age (years)**	1.017 (0.989–1.046)	0.226		
**Menopause**				
No	Reference			
Yes	1.655 (0.783–3.498)	0.187		
**SCCA**	1.007 (0.992–1.022)	0.345		
**Hypertension**				
No	Reference			
Yes	1.109 (0.559–2.199)	0.768		
**Diabetes**				
No	Reference			
Yes	0.802 (0.313–2.054)	0.645		
**AJCC stage**				
I- II	Reference		Reference	
III	2.546 (1.157–5.605)	**0.020**	2.383 (1.043–5.447)	**0.039**
IVA	3.503 (1.596–7.687)	**0.002**	3.352 (1.465–7.672)	**0.016**
**Tumor Volume (cm** ^**3**^ **)**				
< 21.1	Reference		Reference	
≥ 21.1	1.885 (1.022–3.889)	**0.033**	2.973 (1.281–6.902)	**0.011**
**ACCI**				
< 4	Reference		Reference	
≥ 4	3.372 (1.681–6.762)	**0.001**	2.391 (1.148–4.980)	**0.020**
**BMI (kg/m** ^**2**^ **)**	1.039 (0.946–1.140)	0.428		
**PLR**				
< 178.9	Reference			
≥ 178.9	1.925 (0.981–3.777)	0.057		
**PAR**	1.076 (1.053–1.213)	0.081		
**NLR**				
< 3.10	Reference		Reference	
≥ 3.10	2.130 (1.116–4.065)	**0.022**	1.411 (0.695–2.864)	0.341
**LMR**	0.975 (0.876–1.086)	0.649		
**PNI**				
≥ 51.5	Reference			
< 51.5	1.902 (0.897–4.033)	0.097		
**SII**				
< 656.4	Reference		Reference	
≥ 656.4	2.145 (1.088–4.229)	**0.027**	1.028 (0.422–2.500)	0.952
**ALI**				
> 59.2	Reference		Reference	
30.2–59.2	2.704 (0.894–8.183)	0.078	2.641 (0.869–8.028)	0.087
< 30.2	4.196 (1.419–12.409)	**0.010**	8.038 (2.363–27.337)	**0.001**
**SIS**				
0	Reference			
1	1.157 (0.551–2.430)	0.700		
2	1.574 (0.998–5.084)	0.050		

*Abbreviations* ACCI, age-adjusted Charlson comorbidity index; ALI, advanced lung cancer inflammation index, AJCC, American Joint Committee on Cancer; BMI, body mass index; CI, confidence interval; CSCC, cervical squamous cell carcinoma; LMR, lymphocyte-to-monocyte ratio; NLR, neutrophil-to-lymphocyte ratio; OS, overall survival; PAR, platelet-to-albumin ratio; PLR, platelet-to-lymphocyte ratio; PNI, prognostic nutrition index; SCCA, squamous cell carcinoma antigen; SII, systemic immune-inflammation index; SIS, systemic inflammation score

### Construction and validation of nomogram

**Fig. 2:**
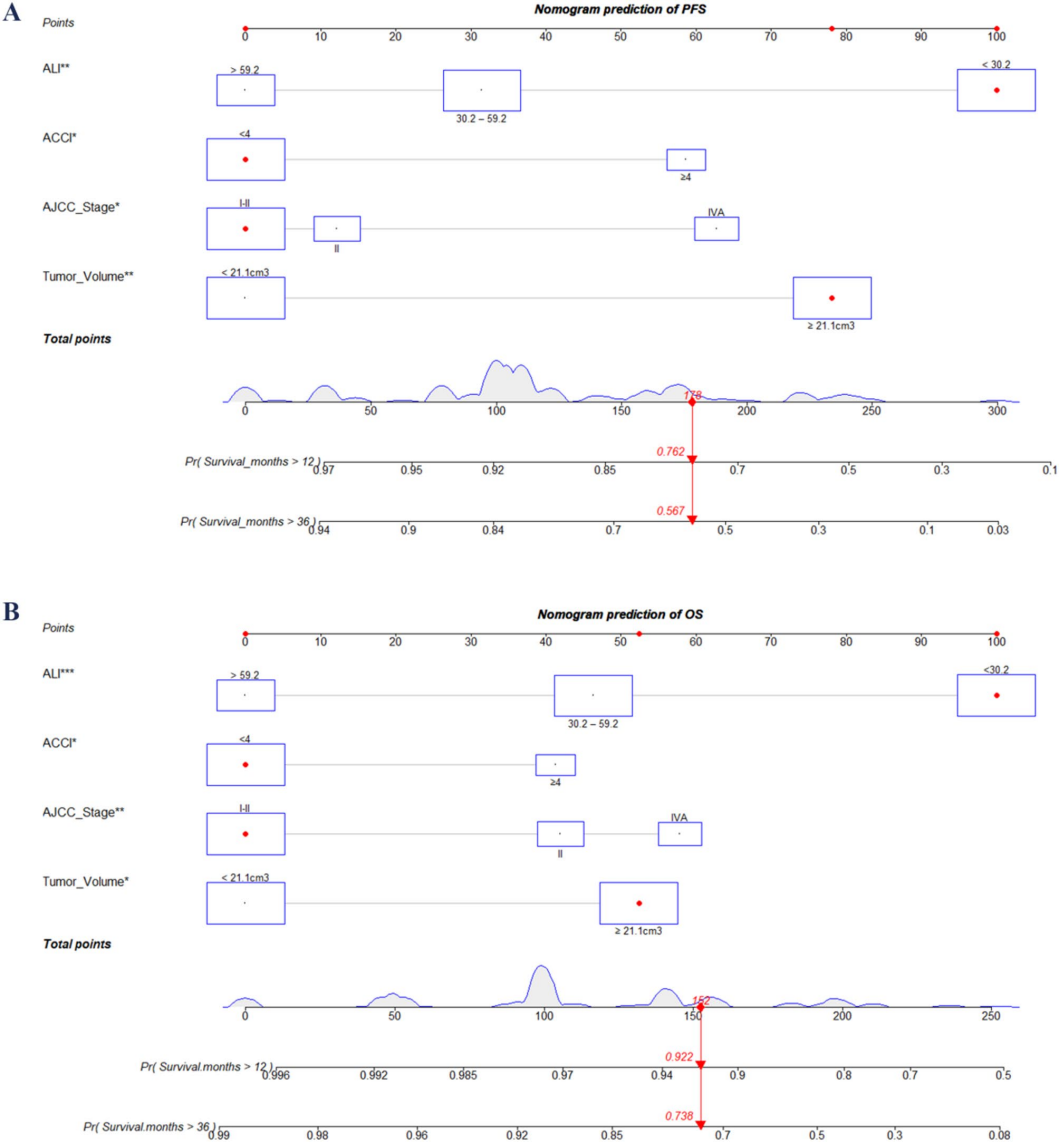
Nomograms predicting PFS (**A**) and OS (**B**) for CSCC who undergoing concurrent chemoradiotherapy. Abbreviations: ACCI, age-adjusted Charlson comorbidity index; ALI, advanced lung cancer inflammation index; AJCC, American Joint Committee on Cancer; OS, overall survival; PFS, progression-free survival

This study constructed nomograms for predicting the 1-year and 3-year PFS and OS of CSCC who undergoing concurrent chemoradiotherapy based on four independent clinical factors (ALI, ACCI, AJCC stage and tumor volume). According to the constructed nomograms, the probabilities of 1-year and 3-year PFS and OS for CSCC who undergoing concurrent chemoradiotherapy can be accurately predicted by calculating the total score of each independent variable (Fig. 2).

The newly developed model demonstrates superior classification performance for samples. The results showed that in the training cohort, the C-index for predicting PFS was 0.743 (95% confidence interval: 0.696–0.790), while in the validation cohort, it was 0.741 (95% confidence interval: 0.694–0.788). Additionally, for the prediction of OS, the C-index in the training cohort was 0.735 (95% confidence interval: 0.686–0.784), and in the validation cohort, it was 0.728 (95% confidence interval: 0.683–0.779) (Table 4).

**Table 4:** The NRI, IDI, and C-index of the nomograms and AJCC stage system for PFS and OS prediction

	Training cohort		*P* value	Validation cohort		*P* value
	Estimate	95% CI		Estimate	95% CI	
**IDI (vs. AJCC** **Stage system)**						
For 1-year PFS	0.122	0.043–0.235	**< 0.001**	0.091	0.029–0.221	**< 0.001**
For 3-year PFS	0.143	0.045–0.281	**< 0.001**	0.096	0.019–0.411	**< 0.001**
For 1-year OS	0.122	0.055–0.290	**< 0.001**	0.132	0.051–0.218	**< 0.001**
For 3-year OS	0.143	0.046–0.284	**< 0.001**	0.150	0.071–0.295	**< 0.001**
**NRI (vs. AJCC** **Stage system)**						
For 1-year PFS	0.396	0.063–0.603	**0.020**	0.244	0.011–0.552	**0.020**
For 3-year PFS	0.326	0.116–0.547	**< 0.001**	0.271	0.019–0.411	**0.040**
For 1-year OS	0.396	0.181–0.629	**< 0.001**	0.312	0.082–0.549	**< 0.001**
For 3-year OS	0.326	0.029–0.511	**< 0.001**	0.279	0.128–0.557	**0.020**
**C-index**						
The nomogram (PFS)	0.743	0.696–0.790		0.741	0.694–0.788	
The nomogram (OS)	0.735	0.686–0.784		0.728	0.683–0.779	
The AJCC Stage (PFS)	0.639	0.592–0.686		0.645	0.559–0.733	
The AJCC Stage (OS)	0.641	0.596–0.686		0.647	0.564–0.723	

*Abbreviations* AJCC, American joint committee on cancer; CI, confidence interval; C-index, concordance index; IDI, integrated discrimination improvement; NRI, net reclassification index; OS, overall survival; PFS, progression-free survival

**Fig. 3:**
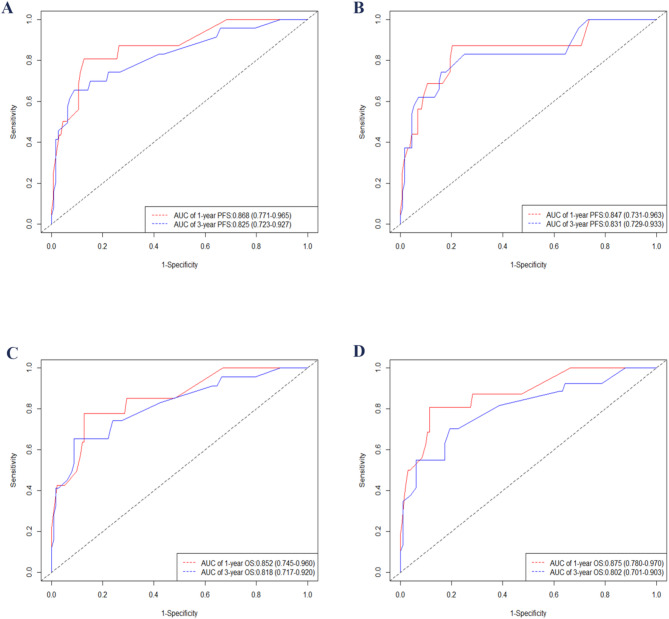
Time-dependent ROC curves for nomograms, displaying AUC values for 1- and 3-year PFS in the training (**A**) and validation (**B**) sets, and 1- and 3-year OS in the training (**C**) and validation (**D**) cohorts. Abbreviations: AUC, area under the curve; OS, overall survival; ROC, receiver operating characteristic; PFS, progression-free survival

Figure 3 illustrates the time-dependent ROC curves of the developed nomograms, thereby validating their satisfactory performance. The discrimination ability of the two nomograms was verified through the ROC curves and their area under the curve (AUC) values (Fig. 3). The results indicated that in the training cohort, the AUCs for predicting 1-year and 3-year PFS were 0.868 and 0.825, respectively (Fig. 3A), while in the validation cohort, they were 0.847 and 0.831, respectively (Fig. 3B). Additionally, for the prediction of OS, the 1-year and 3-year AUCs in the training cohort were 0.852 and 0.818, respectively (Fig. 3C), and in the validation cohort, they were 0.875 and 0.802, respectively (Fig. 3D).

**Fig. 4:**
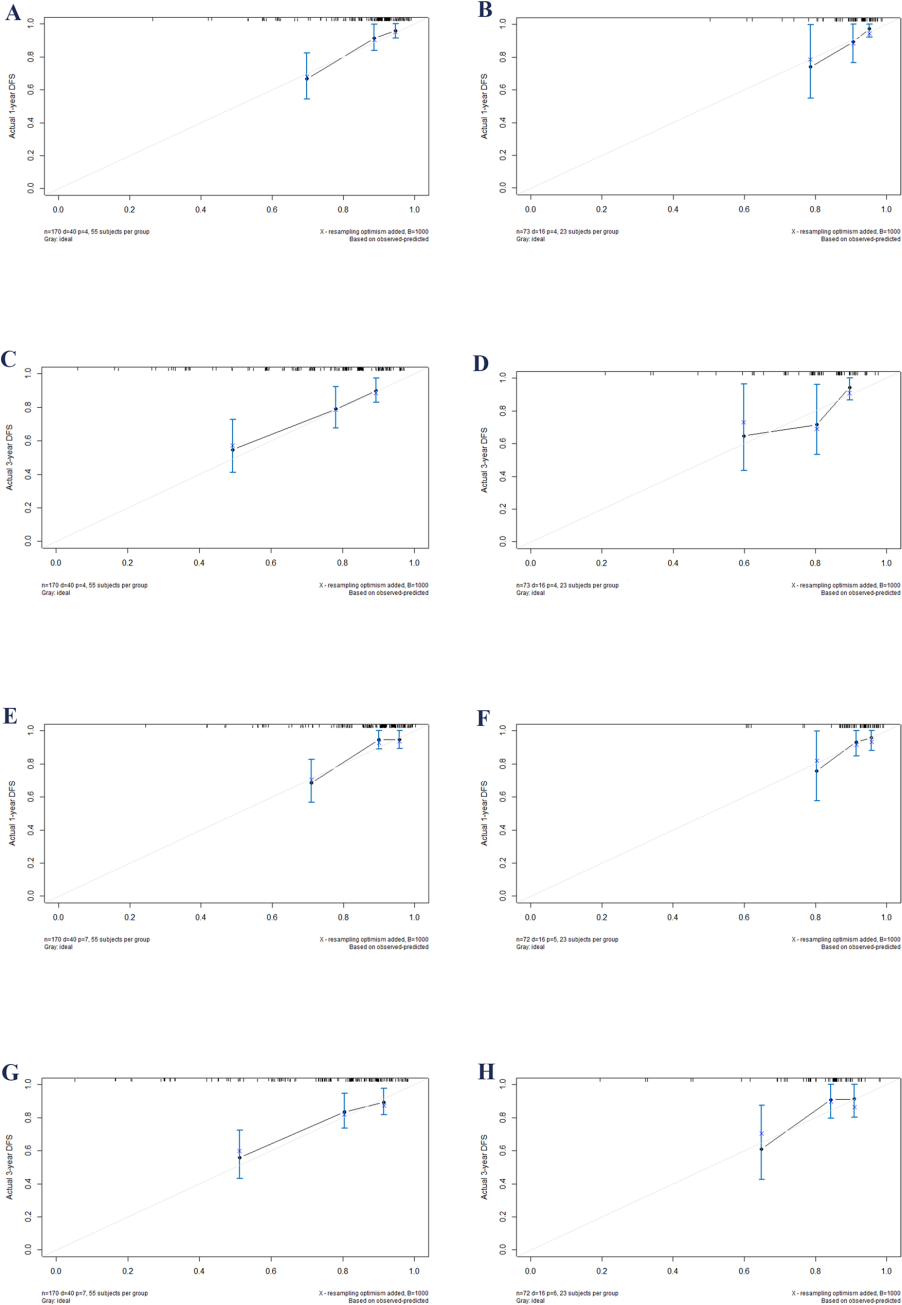
Calibration plots for 1- and 3-year survival in CSCC who undergoing concurrent chemoradiotherapy. Plots **A** and **C** depict 1- and 3-year PFS calibration, while **E** and **G** illustrate OS calibration in the training set. Similarly, plots **B** and **D** show PFS calibration, and **F** and **H** display OS calibration for the validation set. Abbreviations: OS, overall survival; CI, confidence interval; PFS, progression-free survival

**Fig. 5:**
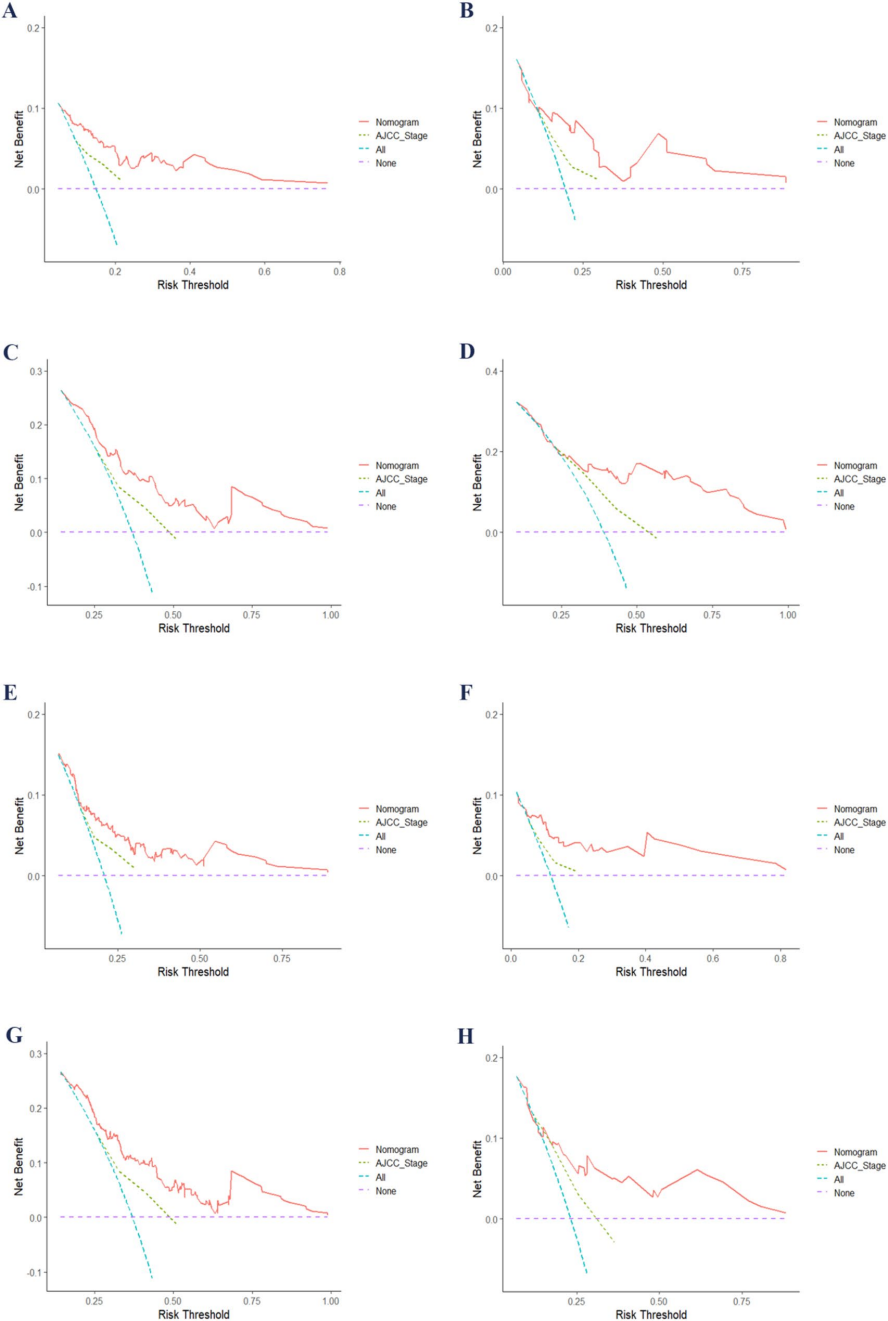
DCA comparing nomograms with AJCC stage for PFS and OS. **A** and **C** show 1- and 3-year PFS DCA curves for the training cohort, while **B** and **D** represent the validation cohort. **E** and **G** display 1- and 3-year OS DCA for the training cohort, with **F** and **H** showing results for the validation cohort. Abbreviations: AJCC, American Joint Committee on Cancer; DCA, decision curve analysis; OS, overall survival

The calibration plot closely approximates the diagonal line, demonstrating a high degree of concordance between the predicted outcomes and the observed data (Fig. 4). DCAs demonstrates that, across the majority of the range, the nomogram outperforms AJCC stage, thereby demonstrating its enhanced clinical utility (Fig. 5).

Furthermore, to evaluate the enhancement of the new model relative to the AJCC staging system, the IDI and NRI were also computed. The IDI assessment results indicated that in the training cohort, the 1-year and 3-year PFS IDIs were 0.122 (95% confidence interval: 0.043–0.235) and 0.143 (95% confidence interval: 0.045–0.281), respectively, while in the validation cohort, they were 0.091 (95% confidence interval: 0.029–0.221) and 0.096 (95% confidence interval: 0.019–0.411), respectively. For OS, the 1-year and 3-year IDIs in the training cohort were 0.122 (95% confidence interval: 0.055–0.290) and 0.132 (95% confidence interval: 0.051–0.218), respectively, and in the validation cohort, they were 0.143 (95% confidence interval: 0.046–0.284) and 0.150 (95% confidence interval: 0.071–0.295), respectively. The NRI assessment results indicated that in the training cohort, the 1-year and 3-year PFS NRI values were 0.396 (95% confidence interval: 0.063–0.603, *P* < 0.001) and 0.326 (95% confidence interval: 0.116–0.547, *P* < 0.001), respectively. In the validation cohort, the corresponding NRI values were 0.244 (95% confidence interval: 0.011–0.552, *P* = 0.020) and 0.271 (95% confidence interval: 0.019–0.411, *P* = 0.040), respectively. For OS, the 1-year and 3-year NRI values in the training cohort were 0.396 (95% confidence interval: 0.181–0.629, *P* < 0.001) and 0.312 (95% confidence interval: 0.082–0.549, *P* < 0.001), respectively. In the validation cohort, the corresponding NRI values were 0.326 (95% confidence interval: 0.029–0.511, *P* < 0.001) and 0.279 (95% confidence interval: 0.128–0.557, *P* = 0.020), respectively. These findings indicate that the inclusion of additional variables in the new nomogram model significantly enhances the performance of the traditional AJCC staging system (Table 4).

### Risk stratification of CSCC who undergoing concurrent chemoradiotherapy

**Fig. 6:**
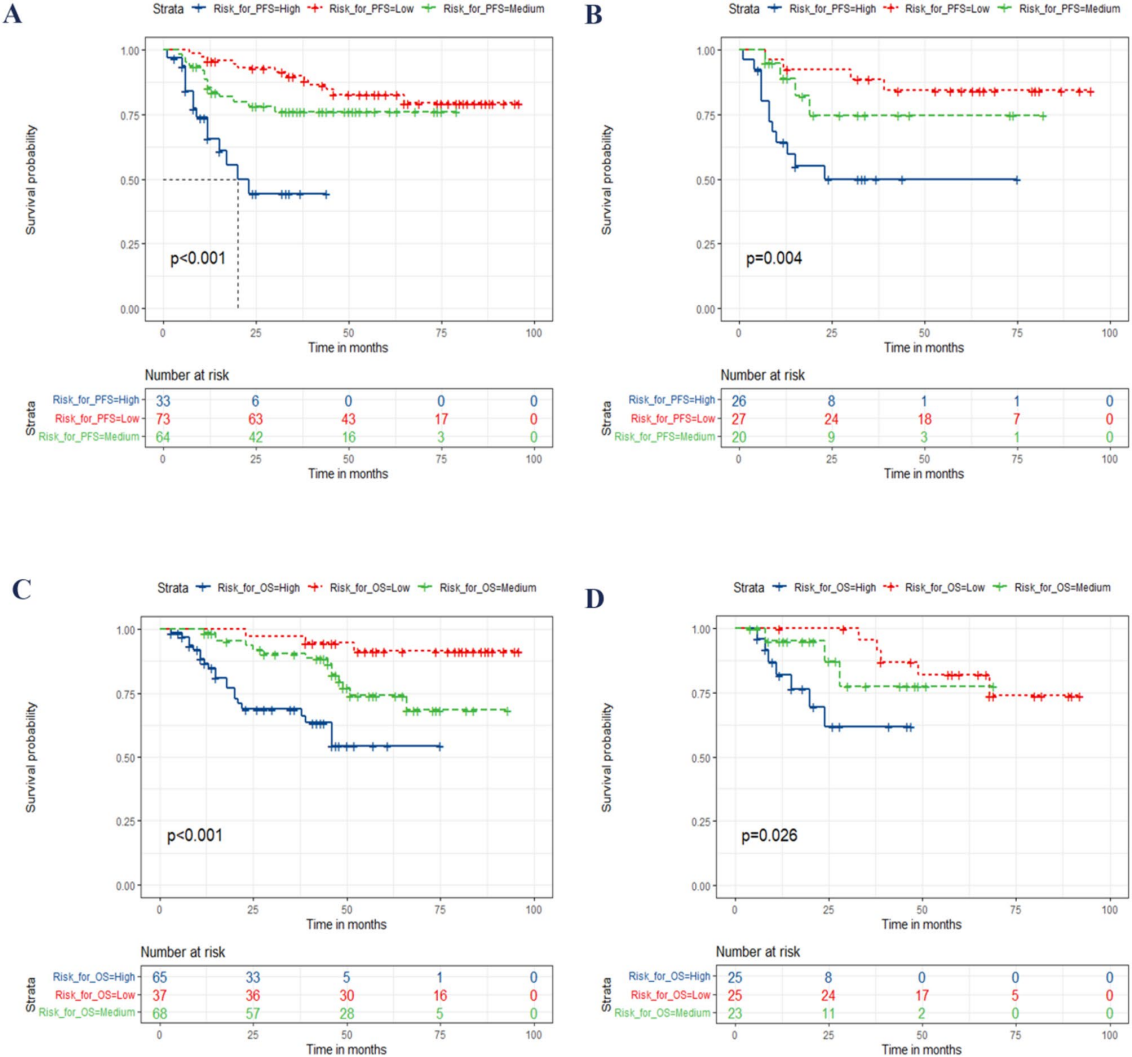
Kaplan-Meier Curves for CSCC who undergoing concurrent chemoradiotherapy in the Training and Validation Cohorts Based on the New Risk Stratification System. Panels **A** and **B** illustrate Kaplan-Meier curves for PFS in the training and validation cohorts, respectively. Panels **C** and **D** depict Kaplan-Meier curves for OS in the training and validation cohorts. Abbreviations: OS, overall survival; PFS, progression-free survival

Based on the total scores calculated from the two nomograms, we stratified the patients into different risk groups to evaluate their PFS and OS. All CSCC who undergoing concurrent chemoradiotherapy were classified into three distinct risk groups according to the total risk points as follows: For PFS prediction: high-risk group (total score ≥ 176.17), medium-risk group (total score 108.36–175.44), and low-risk group (total score ≤ 108.36); For OS prediction: high-risk group (total score ≥ 122.19), medium-risk group (total score 78.88–120.13), and low-risk group (total score ≤ 78.88). Details are shown in Fig. 6. Significant disparities were observed in PFS and OS among patients belonging to different risk subgroups.

## Discussion

Cervical cancer is a relatively common malignant tumor among women worldwide, especially in LMICs, where its incidence and mortality rates remain high [1, 7]. As the most populous country in the world, China is burdened with a heavy load of cervical cancer. Although the prevalence of HPV vaccines and cervical cancer screening has reduced the incidence of cervical cancer to some extent in recent years [6, 23], due to the uneven distribution of medical resources and insufficient screening coverage, cervical cancer remains a major public health challenge in China [24–26]. SCC is the most common histological type of cervical cancer, and concurrent chemoradiotherapy is the main treatment for cervical squamous cell carcinoma. Currently, studies on the prognosis of cervical cancer mainly focus on factors related to tumor staging [11], such as tumor size [27], lymph node [28, 29], and imaging features [30, 31].

However, limited research has been conducted on inflammatory markers as prognostic indicators for CSCC patients undergoing concurrent chemoradiotherapy. Notably, there is a lack of a specific research assessing the impact of ALI on the prognosis of these patients. Therefore, it is imperative to further investigate this issue and evaluate the prognostic significance of ALI in patients with CSCC receiving concurrent chemoradiotherapy. Additionally, the most commonly used tool for estimating prognosis remains the Tumor, Node, Metastasis (TNM) staging system, with the latest version being the 9th edition of the AJCC staging system. Since staging systems are primarily based on anatomical parameters, many important clinical variables that influence prognosis are often overlooked [32]. Consequently, this study aims to incorporate several critical clinical variables into the prognostic model to enhance the predictive accuracy of the traditional staging system and provide guidance for personalized treatment.

Tumor volume is a clinical indicator associated with tumor staging and is recognized as a critical prognostic factor in cervical cancer. Jingjing Zhang et al.‘s study [33] demonstrates that tumor volume has significant predictive value for the prognosis of high-risk patients, with larger tumor volumes correlating with poorer patient outcomes. Additionally, Aaron E. Wagner et al.‘s research [27] confirms that patients with larger tumors exhibit significantly worse risk ratios. Our research also found that tumor volume is a significant factor influencing the PFS and OS of CSCC patients who have undergone concurrent chemoradiotherapy. The larger the tumor volume, the worse the PFS and OS of the patients.

As an inflammatory mediator, the impact of ALI on the prognosis of various malignant tumors has emerged as a focal point of research in recent years. Multiple studies have shown that ALI is the best inflammatory biomarker for overall survival rate in lung cancer patients [16], The study indicates that in patients with lung cancer, a lower ALI is associated with a higher risk of death. Qian Li et al. [34] established a Nomogram model including ALI and accurately predicted the success rate of immunotherapy for patients with advanced liver cancer, this study indicates that for patients with hepatocellular carcinoma receiving immunotherapy, the optimal cut-off value of ALI is 36.5. The median OS of patients with ALI ≤ 36.5 is shorter than that of patients with ALI > 36.5. The research [35] by Taichi Horino et al. indicates that there is no consensus on the optimal cutoff value of ALI at present, and its value may vary significantly due to factors such as cancer type, tumor stage, and patient gender. A meta-analysis [36] incorporated 18 studies involving 6898 patients. The aggregated findings indicated that a lower ALI was associated with worse OS (HR = 1.914, 95% CI: 1.514–2.419, *P* < 0.001), disease-free survival (DFS) (HR = 1.631, 95% CI: 1.197–2.224, *P* = 0.002), and PFS (HR = 1.679, 95% CI: 1.073–2.628, *P* = 0.023) in patients with gastrointestinal malignancies. In this study, for CSCC patients who have undergone concurrent chemoradiotherapy, those with low ALI had poorer PFS and OS, a result consistent with previous studies, indicating that ALI is of significant value in assessing the prognosis of such patients.

ACCI is an index that comprehensively assesses comorbidity and age, and it has also been reported to predict the prognosis of various cancers [37–39]. To date, no studies have explored the association between ACCI and CSCC patients who have undergone concurrent chemoradiotherapy. This study is the first to incorporate ACCI into the analysis, and the results show that patients with an ACCI value ≥ 4 have significantly worse PFS and OS than those with an ACCI value < 4, and the difference is statistically significant.

The strength of the nomogram lies in its intuitiveness and simplicity. It visually represents the relationship between variables through geometric figures, enabling users to rapidly obtain results without complex calculations. This tool is especially suited for scenarios requiring rapid decision-making, as it conserves time and effort while effectively managing the influence of multiple variables. Consequently, it enhances the intuitiveness and efficiency of data visualization [12, 13, 40]. In recent years, nomograms, as multivariable prediction models, have gained widespread application in oncology owing to their superior predictive accuracy [13, 41–48]. Wing-Keen Yap et al.‘s research [49] indicates that the nomogram (with web-based tool) can be useful for assessing the probability of survival at 3, 6, and 12 months in patients with metastatic lung cancer referred for radiotherapy to treat bone metastases. In the research of cervical cancer, the application of nomograms mainly focuses on factors related to tumor staging [11, 28, 29, 31, 32, 50, 51], while studies on inflammatory indicators are relatively scarce [52, 53]. Wang Shanshan et al.‘s research [54] indicates that the prognostic nomogram incorporating nutritional-inflammation indicators significantly enhances the prediction accuracy of long-term outcomes for cervical cancer patients receiving adjuvant radiotherapy. Hu Jing et al. [55] developed a prognostic nomogram for cervical cancer patients undergoing radiotherapy, integrating clinical parameters, inflammatory markers, and the occurrence of acute radiation enteritis. External validation confirmed that inflammatory markers hold significant predictive value for the prognosis of cervical cancer. These studies indicate that certain inflammatory markers have a significant impact on prognosis. However, the potential association between ALI and cervical cancer prognosis remains unclear, despite its importance as a validation marker. Consequently, this study serves as a crucial addition to existing research.

Although this study developed a prognostic nomogram based on multiple independent clinical factors such as ALI and ACCI and evaluated its clinical utility, there are still many deficiencies. The sample size is limited and the data source is single, which affects the universality and stability of the results; potential influencing factors such as HPV infection status were not included, nor was the impact of treatment compliance on prognosis explored; as a retrospective study, there are data biases and confounding factors, and model validation and external validation are insufficient; the biological mechanism was not explored, the heterogeneity of treatment regimens was not considered, the risk stratification criteria need to be optimized, and the quality of life of patients was not evaluated. Future improvements are needed in these aspects to enhance the clinical application value of the model.

## Conclusion

This study developed two ALI-based nomograms to predict the PFS and OS of CSCC who undergoing concurrent chemoradiotherapy. The newly developed model demonstrates superior performance compared to the AJCC staging system.

## Electronic supplementary material

Below is the link to the electronic supplementary material.

Supplementary Material 1

Supplementary Material 2

Supplementary Material 3

## Data Availability

No datasets were generated or analysed during the current study.

## Abbreviations

ACCI: Age-adjusted Charlson comorbidity index
AJCC: American Joint Committee on Cancer
ALI: Advanced lung cancer inflammation index
BMI: Body mass index
CI: Confidence interval
C-index: Concordance index
DCA: Decision curve analysis
ECOG PS: Eastern Cooperative Oncology Group performance status score
FIGO: International Federation of Gynecology and Obstetrics
HIV: Human Immunodeficiency Virus
HPV: Human papilloma virus
HSCC: Hypopharyngeal squamous cell carcinoma
IDI: Integrated discrimination improvement index
IQR: Interquartile range
LMICs: Low- and Middle-Income Countries
LMR: Lymphocyte-to-monocyte ratio
NSCLC: Non-small cell lung cancer
NLR: Neutrophil-to-lymphocyte ratio
OS: Overall survival
PAR: Platelet-to-albumin ratio
PFS: Progression-free survival
PLR: Platelet-to-lymphocyte ratio
PNI: Prognostic nutrition index
ROC: Receiver operating characteristic curves
SCCA: Squamous cell carcinoma antigen
SII: Systemic immune-inflammation index
SIS: Systemic inflammation score
TNM: Tumor, Node, Metastasis
VIF: Variance inflation factor
