# Supplementary material for: Nomogram based on the advanced lung cancer inflammation index and other relevant clinical factors for patients with cervical squamous cell carcinoma undergoing concurrent chemoradiotherapy
Source: BMC Cancer. 2025 Jul 1;25:1043. doi: 10.1186/s12885-025-14465-6 (PMC12210734; doi:10.1186/s12885-025-14465-6)
Supplement: Supplementary file 1 — Supplementary Material 1 [file 12885_2025_14465_MOESM1_ESM.docx]

| **Table S1**. Calculation formulas for the research. | |
| --- | --- |
| **Clinical index** | **Calculation formula** |
| PLR | Platelet count / lymphocyte count |
| PAR | Platelet count / albumin (g/L) |
| NLR | Neutrophil count / lymphocyte count |
| LMR | Lymphocyte count / monocyte count |
| PNI | Albumin (g/L) + (lymphocyte count ×5) |
| SII | Platelet count × NLR |
| SIS | Albumin < 40 g/L & LMR < 4.44 is scored as 2; Albumin ≥ 40 g/L or LMR ≥ 4.44 is scored as 1; Albumin ≥ 40 g/L & LMR ≥ 4.44 is scored as 0 |
| BMI | Mass (kg) / height^2^(m^2^) |
| ALI | Albumin × BMI / NLR |
| *Abbreviation* ALI, advanced lung cancer inflammation index; BMI, body mass index; LMR, lymphocyte-monocyte ratio; NLR, neutrophil-to-lymphocyte ratio; PLR, platelet-to-lymphocyte ratio; PNI, prognostic nutrition index; SII; systemic immune-inflammation index, SIS, systemic inflammation score. | |
