# Supplementary material for: Nomogram based on the advanced lung cancer inflammation index and other relevant clinical factors for patients with cervical squamous cell carcinoma undergoing concurrent chemoradiotherapy
Source: BMC Cancer. 2025 Jul 1;25:1043. doi: 10.1186/s12885-025-14465-6 (PMC12210734; doi:10.1186/s12885-025-14465-6)
Supplement: Supplementary file 2 — Supplementary Material 2 [file 12885_2025_14465_MOESM2_ESM.docx]

| **Table S2**. Age-adjusted Charlson comorbidity index. | |
| --- | --- |
| **Scores** | **Clinical conditions** |
| **For comorbidities** |  |
| 1 point | Cerebrovascular disease; Chronic pulmonary disease; Connective tissue disease; Congestive heart failure; Dementia; Diabetes; Mild liver disease; Myocardial infarction; Peripheral vascular disease; Ulcer disease |
| 2 points | Any tumor; Diabetes with endo organ damage; Hemiplegia; Leukemia; Lymphoma; Moderate or severe renal disease |
| 3 points | Moderate or severe liver disease |
| 6 points | Acquired immune deficiency syndrome; Metastatic solid tumor |
| **For age** |  |
| 1 (up to 4 points) | Each decade over age 40 years |
