# Supplementary material for: Nomogram based on the advanced lung cancer inflammation index and other relevant clinical factors for patients with cervical squamous cell carcinoma undergoing concurrent chemoradiotherapy
Source: BMC Cancer. 2025 Jul 1;25:1043. doi: 10.1186/s12885-025-14465-6 (PMC12210734; doi:10.1186/s12885-025-14465-6)
Supplement: Supplementary file 3 — Supplementary Material 3 [file 12885_2025_14465_MOESM3_ESM.docx]

| **Table S3** Multicollinearity Detection Among Prognostic Variables in HSCC Patients Undergoing Radiotherapy | | |
| --- | --- | --- |
|  | **Tolerance** | **VIF** |
| Age | 0.318 | 3.149 |
| Menopause | 0.340 | 2.944 |
| Pathological | 0.886 | 1.129 |
| Stage | 0.595 | 1.679 |
| AJCC_Stage | 0.648 | 1.544 |
| SCCA | 0.795 | 1.257 |
| Tumor_Volume | 0.709 | 1.411 |
| Chemotherapy | 0.834 | 1.199 |
| ACCI | 0.609 | 1.642 |
| BMI | 0.939 | 1.065 |
| PLR | 0.435 | 2.298 |
| PAR | 0.707 | 1.415 |
| NLR | 0.472 | 2.119 |
| LMR | 0.542 | 1.844 |
| PNI | 0.652 | 1.533 |
| SII | 0.336 | 2.975 |
| SIS | 0.605 | 1.653 |
| ALI | 0.532 | 1.880 |
| *Abbreviations* ACCI, age-adjusted Charlson comorbidity index; ALI, advanced lung cancer inflammation index, AJCC, American Joint Committee on Cancer; BMI, body mass index; HSCC, hypopharyngeal squamous cell carcinoma; LMR, lymphocyte-to-monocyte ratio; NLR, neutrophil-to-lymphocyte ratio; PAR, platelet-to-albumin ratio; PLR, platelet-to-lymphocyte ratio; PNI, prognostic nutrition index; SCCA, squamous cell carcinoma antigen; SII, systemic immune-inflammation index; SIS, systemic inflammation score; VIF, variance inflation factor. | | |
